# Poor Physical Fitness Performance as a Predictor of General Adiposity in Taiwanese Adults

**DOI:** 10.3390/ijerph17082686

**Published:** 2020-04-14

**Authors:** Yi-Tien Lin, Po-Fu Lee, Tian-Shyug Lee, Chien-Chang Ho

**Affiliations:** 1 Graduate Institute of Business Administration, Fu Jen Catholic University, New Taipei City 242, Taiwan; sweet.oc18@gmail.com (Y.-T.L.); 036665@mail.fju.edu.tw (T.-S.L.); 2 Graduate Institute of Sport Coaching Science, Chinese Culture University, Taipei City 111, Taiwan; 3 Department of Physical Education, Fu Jen Catholic University, New Taipei City 242, Taiwan; 4 Artificial Intelligence Development Center, Fu Jen Catholic University, New Taipei City 242, Taiwan; 5 Research and Development Center for Physical Education, Health, and Information Technology, Fu Jen Catholic University, New Taipei City 242, Taiwan

**Keywords:** exercise capacity, body mass index, overweight, obesity

## Abstract

The purpose of the present study was to investigate the association between different levels of health-related physical fitness measurements and obesity status in Taiwanese adults. A cross-sectional study was conducted by reviewing the National Physical Fitness Survey in Taiwan (HPFSIT) database. Responses from 60,056 participants, aged 23–64 years from the database were collected in the present study. Data from a standardized structured questionnaire and health-related physical fitness tests were analyzed. The quartiles of each physical fitness measurement were used for unconditional logistic regression analyses. Our results indicated clear trends in the association between cardiorespiratory fitness and overweight/obesity. Overweight and obesity were associated with a 10% to 60% increased risk of low levels of cardiorespiratory fitness in men and a 10% to almost 30% increased risk in women. However, the association between muscle strength/endurance and obesity status as well as flexibility and obesity status needs further investigation.

## 1. Introduction

Today, facing the challenges caused by non-communicable diseases (NCDs) is crucial. Obesity has been reported as one of the risk factors for NCDs, particularly for chronic diseases [1]. According to the World Health Organization [2], in 2016, 39% of adults aged 18 years and over were considered to be overweight and 13% of them were obese. Research has indicated that the increase in the prevalence of obesity over the past three decades has increased the risk for cardiovascular and cancer deaths [3,4,5]. Obesity is considered an antecedent in the development of NCDs [6].

Weight gain and obesity are primarily due to excess energy intake and insufficient energy expenditure. However, determining human body weight is not just a matter of measuring energy in and out. From a general perspective, individual total energy expenditure (TEE) consists of resting energy expenditure (REE), diet-induced energy expenditure (DEE), and activity-induced energy expenditure (AEE) [7]. These expenditure processes are influenced by many individual factors, such as genetic variability [8,9], physiological responses [10], built environment [11], and social environment [12]. Nevertheless, it is likely that there are other processes that are still unidentified. For example, studies have increased physical activity participation and reduced sedentary behavior among the general population, but the results have revealed considerable variability [13,14]. In addition, even among individuals living in a controlled environment, results regarding energy expenditure are still variable [15]. Also, the predictors for responses to a given intervention remain elusive [8,16]. 

Physical fitness is one of the common predictors for obesity. Research has suggested that individual fitness levels can effectively attenuate the health-related impacts of obesity [17,18,19]. Individual fitness levels may be more important than body weight in terms of maintaining good health status. For example, research has indicated that lower cardiorespiratory fitness is related to higher premature mortality in individuals with normal weight, overweight, and obesity [19]. In fact, this so-called “obesity paradox” first presented itself decades ago [20]. A similar phenomenon has been observed in the relationship between obesity and cardiovascular disease (CVD) [21], coronary heart disease (CHD) [22], and total mortality [23]. Further, although higher levels of adiposity are associated with higher mortality, better cardiovascular fitness has been found to attenuate this association [24]. Lifestyle modification, specifically, changes in diet, physical activity, and exercise are fundamental to obesity management [25]. Maintaining proper fitness levels may be more critical than pursuing a lean body. However, the relationship between physical fitness and obesity is complex and multifactorial, thus further investigation is needed [26]. To the best of our knowledge, the relationship between different levels of physical fitness and obesity is less discussed in the litierature. Little is known of the relationship between muscle strength and obesity, as well as flexibility and obesity.

Therefore, the purpose of this study was to investigate the association between different levels of health-related physical fitness measurements and obesity status in Taiwanese adults.

## 2. Materials and Methods 

### 2.1. Study Design and Participants

The database from the National Physical Fitness Survey in Taiwan (NPFSIT) was utilized for this cross-sectional study. The Sports Administration, Ministry of Education in Taiwan, governs the NPFSIT. The details of the design, sampling protocols, and data validation in the NPFSIT have been described previously, and the de-identified data have been released for research purposes [27]. Participants were recruited from a total of 46 physical fitness test stations in 22 cities and counties in Taiwan from October 2014 and March 2015. Informed consent was obtained from the participants after they were given a full explanation of the NPFSIT. Furthermore, the design and analysis protocol of the present study was approved by the Institutional Review Board of Fu Jen Catholic University in Taiwan (FJU-IRB C108006). A total of 60,056 participants (29,166 men and 30,890 women) aged 23 to 64 years were included in this analysis.

### 2.2. Data Collection

All of the NPFSIT assessments were conducted by qualified, certified examiners who had attended a regional training seminar, as previously reported [27,28]. Three phases—a structured questionnaire survey, preliminary safety screens, and physical fitness tests—comprised the data collection protocol of the survey. The data for participants’ sociodemographic characteristics (such as age, gender, education, monthly income, and marital status), lifestyle including smoking, betel nut chewing, and diet habits, and perceived health status were collected. Education status was divided into three levels (elementary school or lower, junior or senior school, and college or higher). Monthly income was divided into less than 20,000 NTD, 20,001 to 40,000 NTD, and more than 40,001 NTD. Marital status was divided into married, never married, and divorced/separated/widowed. In terms of smoking and betel nut chewing habits, participants were listed as never-users, former-users, and current users. Perceived health status was divided into excellent, good, fair, very bad and poor. 

After the questionnaire survey, participants were assisted in measuring their resting heart rate and blood pressure for a preliminary safety check before physical fitness tests were conducted. Participants with systolic blood pressure exceeding 140 mmHg, or diastolic blood pressure exceeding 90 mmHg, and those with heart disease, hypertension, chest pain, vertigo, or musculoskeletal disorders were excluded. 

### 2.3. Anthropometrics and Obesity Status

Anthropometric variables such as body weight, height, waist circumference (WC), and hip circumference (HC) were first measured in the NPFSIT. WC and HC measurements (measured to the nearest 0.1 cm) were performed twice, and the mean value was used. WC was measured at the midway between the lowest rib and iliac crest, after a normal outbreath. HC was measured at the site of the largest convexity of the buttocks below the hip plates.

Then, the body mass index (BMI, kg/m^2^) and waist-to-hip ratio (WHR) were calculated. The participants were classified into normal weight, overweight, and obesity groups in accordance with the cut-off points suggested by the Health Promotion Administration, Ministry of Health and Welfare in Taiwan. A BMI between 18.5 and 24 kg/m^2^ was considered normal weight, between 24 and 27 kg/m^2^ was considered as overweight, and exceeding 27 kg/m^2^ as obesity [29].

### 2.4. Measures of Health-Related Physical Fitness

The following tests of physical fitness were conducted: cardiorespiratory fitness was measured via the 3-min step test [28], muscle strength/endurance via the 1-min sit-up test (reps/min) [30], and flexibility via the sit-and-reach test (cm) [31]. Only the sit-and-reach test needed to be performed twice, and the best result was applied.

Participants were asked to avoid any other physical activity before these tests. A 10-min warm-up was introduced by the examiner and the participant did this before the physical fitness assessment. All participants performed the tests in the following order with a sufficient break period (3–5 min) between tests: 1-min sit-up test, 2-min step test, sit-and-reach test, and 3-min step test. 

### 2.5. Statistical Analyses

Data were analyzed using SAS 9.4 (SAS Institute, Cary, NC, USA). The student’s *t*-test was performed to analyze continuous variables, and the chi-square test was used for the analysis of categorical variables. Unconditional logistic regression analyses were conducted to evaluate the linear association among cardiorespiratory fitness, muscle strength/endurance, or flexibility, and obesity status. All regression models were adjusted for age, WHR, education, monthly income, marital status, self-reported health status, smoking status, and chewing betel nuts. Then, the adjusted odds ratios (ORs) with 95% confidence intervals (CIs) were calculated. In order to examine the dose–response relationship between physical fitness performance and obesity status, four different categories (quartiles) were applied for each physical fitness measurement according to gender. The low quartile was composed of participants who had the best results in each physical fitness measurement, and it was assigned as the reference group for further analysis. Values are presented as means ± standard deviation (SD) or frequency percentages. Statistical results were significant with *p* < 0.05. 

## 3. Results

Table 1 and Table 2 show the anthropometric measurements and demographic characteristics of the participants according to BMI status in Taiwanese adults. Data from 29,166 men and 30,890 women participants were used in the study. Variability among groups were found in all of the characteristic variables for both genders. Table 3 presents the χ^2^ test results for each physical fitness test among BMI groups. Generally, the normal weight population performed significantly better than the overweight population in the 3-min step test, 1-min sit-up test, and sit-and-reach test, followed by the obese population. This result remained consistent for both men and women.

Table 4 shows the results of the logistic regression analyses for overweight risk for different physical fitness tests and their performance levels. Participants were divided into four levels in accordance with their performance (quartiles). Regression models were adjusted by the potential confounding factors (e.g., age, WHR, education, monthly income, marital status, self-reported health status, smoking status, and chewing betel nuts). Our results show a clear trend in cardiorespiratory fitness. They indicate a reversed relationship between overweight risk and 3-min step test performance. The ORs ranged from 1.11 to 1.24 for men and from 1.17 to 1.76 for women.

Analysis of the 1-min sit-up test exhibited a similar trend, but the relationship was minor for women (ORs ranged from 1.207 to 1.361). Surprisingly, a reduced OR was found in the lowest level of 1-min sit-up performance in men (OR: 0.896). Additionally, two reduced ORs were found in the men’s sit-and-reach tests. The association between women’s sit-and-reach test and the risk of being overweight fluctuated (OR: 1.118 and 0.835).

Similarly, Table 5 presents the results of the logistic regression analyses for obesity risk. Reversed relationships between obesity risk and cardiorespiratory fitness levels were also found. The ORs ranged from 1.140 to 1.637 in men, and 1.538 to 2.855 in women, and followed the trend (*p* < 0.001). Furthermore, the results of the 1-min sit-up test remained positive in both men and women. Only minor correlations were observed in men (ORs from 1.174 to 1.265), but for women, they were considerable (ORs from 1.459 to 2.387). In terms of the results for the sit-and-reach levels, only one reduced OR (0.795) was found for men; again, this fluctuated for women (OR: 1.198 and 0.843). 

## 4. Discussion

The purpose of the present study was to investigate the association between physical fitness and obesity status. A representative database was used for this cross-sectional study. Our results indicate a correlation between low levels of cardiorespiratory fitness and unhealthy body weight status. In particular, individuals with overweight or obesity problems were found to have a 1.1 to 1.6 times higher risk of low levels of cardiorespiratory fitness for men, with a 1.1 to almost 3 times higher risk for women. As expected, this result is supported by recent research [32].

Another study investigated the predictive effects of cardiorespiratory fitness on future BMI in Canada [33]. This was a 20-year longitudinal study, and the results indicated that low levels of cardiorespiratory fitness were associated with a higher future risk of obesity. Women with better cardiorespiratory fitness were at a lower risk of experiencing a weight gain of 10 kg or more in the years following the study. Further, improved cardiorespiratory fitness over time can reduce age-related weight gain [34]; otherwise, individuals with lower cardiorespiratory fitness, particularly men, might increase their chances of being overweight or obese [35]. Hence, cardiorespiratory fitness seems to moderate BMI over time. This is probably associated with resting metabolic rate (RMR). Specifically, research has indicated a correlation between cardiorespiratory fitness and RMR [36], which is fundamental for daily TEE regulation, and thus it attenuates the risk of being obese as well as the storage of adiposity.

In contrast, our results indicated that women with lower muscle strength/endurance are more likely to have obesity. The same results have been previously presented as a negative correlation between abdominal muscle strength and BMI in women [37,38]. However, this association with obesity was minor in men. Additionally, an unexpected decreasing risk was found for being overweight. This result seems to be affected by adjustment for confounders. Significant negative associations were found in men before model adjustment (ORs for overweight: 1.25–1.54; ORs for obesity: 1.61–2.48). For example, fat distribution has been known to change with age [39]. Future studies may focus on the moderating or mediating effects of these confounders. Furthermore, fatty infiltration of muscle and central adiposity is associated with reduced strength [40,41]. Thus, based on this, the confounders we used in this study, such as age and WHR, may contribute to eliminating the association between sit-up test results and obesity status. Future studies are suggested to investigate the mediating and moderating effects of potential factors on the muscle strength/endurance–obesity relationship.

Unlike the measurements mentioned above, the results for flexibility in both men and women are elusive. Notably, the association between overweight and obesity and flexibility was reduced in men. This result implies that men who performed less well in the sit-and-reach test are no more likely to be overweight or obese. Additionally, the results for women showed fluctuations in the correlation between overweight or obesity and flexibility. To the best of our knowledge, the only research we can find on this did not identify any significant relationship between flexibility and obesity [42,43]. A possible explanation for this inconsistency may be the classification of flexibility levels, which has not been discussed previously. However, the mechanism remains unclear. Future research should focus on the relationship between obesity and flexibility-related factors, such as musculoskeletal biomechanics, functional degeneration of articular capsule and ligament, and stiffness of muscle-tendon or connective tissue.

The strength of the present study was the use of a representative database for analysis. However, some limitations should be addressed. First, the research population was composed of 23- to 64-year-old Taiwanese adults. Future studies should focus on participants from different age groups, of different races, and of different cultures (lifestyles). Second, the present study aimed to understand the association between different physical fitness performances and obesity status. However, based on the limitations stemming from the fact that it is a cross-sectional study, we suggest future studies should be conducted with a longitudinal design to obtain a complete understanding of this matter.

## 5. Conclusions

In conclusion, according to the results of this study, two physical fitness measurements clearly predicted the risk of overweightness and obesity. Specifically, cardiorespiratory fitness and muscular strength provided considerable information on overweight and obesity risks in both genders. However, further research on the association between flexibility and obesity status is still needed.

## Figures and Tables

**Table 1:** Anthropometric measurements of the study participants.

Variables	Men (n = 29,166)				Women (n = 30,890)			
	Obesity (n = 6947)	Overweight (n = 10,141)	Normal Weight (n = 12,078)	*p*	Obesity (n = 3918)	Overweight (n = 6666)	Normal Weight (n = 20,306)	*p*
Age (years)	41.28 ± 11.01	42.05 ± 11.33	38.99 ± 11.88	< 0.001 *	45.50 ± 11.71	46.21 ± 11.42	41.65 ± 11.54	<0.001 *
Body weight (kg)	84.60 ± 7.53	73.96 ± 5.95	64.45 ± 6.21	< 0.001 *	72.75 ± 7.35	62.73 ± 5.07	53.89 ± 4.97	<0.001 *
Height (cm)	170.22 ± 6.13	170.56 ± 6.30	171.00 ± 6.42	< 0.001 *	156.98 ± 5.87	157.38 ± 5.84	158.69 ± 5.59	<0.001 *
BMI (kg/m^2^)	29.18 ± 1.84	25.39 ± 0.85	22.01 ± 1.40	< 0.001 *	29.49 ± 2.17	25.29 ± 0.86	21.38 ± 1.45	<0.001 *
WC (cm)	94.16 ± 6.42	85.99 ± 5.40	78.39 ± 5.87	< 0.001 *	89.26 ± 7.54	80.93 ± 6.02	72.59 ± 6.13	<0.001 *
HC (cm)	103.37 ± 5.03	97.78 ± 4.31	92.91 ± 4.45	< 0.001 *	104.00 ± 5.71	97.69 ± 4.59	91.89 ± 4.60	<0.001 *
WHR	0.91 ± 0.05	0.88 ± 0.05	0.84 ± 0.05	< 0.001 *	0.86 ± 0.06	0.83 ± 0.06	0.79 ± 0.06	<0.001 *

Abbreviations: Values are expressed as means ± standard deviation. * *p* < 0.05.

**Table 2:** Demographic characteristics of the study participants.

Variables	Men (n = 29,166)				Women (n = 30,890)			
	Obesity (n = 6947)	Overweight (n = 10,141)	Normal Weight (n = 12,078)	*p*	Obesity (n = 3918)	Overweight (n = 6666)	Normal Weight (n = 20,306)	*p*
Education level (%)				< 0.001 *				<0.001 *
Elementary school or lower	2.30	1.78	1.36		9.90	8.21	3.84	
Junior or senior school	26.73	24.80	22.01		39.49	37.69	28.85	
College or higher	70.97	73.43	76.63		50.61	54.10	67.31	
Income level (%)				<0.001 *				<0.001 *
≦20,000 NTD	14.04	12.65	16.87		33.08	30.42	24.08	
20,001–40,000 NTD	34.40	33.08	35.73		45.92	44.14	47.89	
≧40,001 NTD	51.56	54.27	47.40		20.99	25.44	28.03	
Marital status (%)				<0.001 *				<0.001 *
Never married	54.36	53.39	51.65		57.78	57.01	54.87	
Married	42.78	44.17	46.46		35.54	36.43	40.64	
Divorced/separation/widowed	2.86	2.44	1.89		6.69	6.55	4.49	
Self-reported health status (%)				<0.001 *				<0.001 *
Excellent or good	53.97	64.82	64.98		51.25	59.78	61.18	
Fair	36.09	30.09	29.92		37.95	33.11	32.88	
Very bad or poor	9.94	5.08	5.09		10.80	7.10	5.94	
Smoking status (%)				<0.001 *				0.005 *
Never	65.82	69.92	73.48		94.52	95.84	95.88	
Current	22.67	19.22	18.09		3.85	2.88	2.95	
Former	11.50	10.85	8.44		1.63	1.28	1.17	
Chewing betel nuts				<0.001 *				<0.001 *
Never	86.79	90.53	92.70		97.58	98.86	99.25	
Current	5.41	3.28	2.57		2.17	0.79	0.52	
Former	7.79	6.19	4.73		0.24	0.35	0.23	

Abbreviations: NTD, New Taiwan Dollar; Values are expressed as means ± standard deviation. * p < 0.05.

**Table 3:** Health-related physical fitness and anthropometric characteristics measurements according to general obesity status in Taiwanese adults.

Variables	Obesity	Overweight	Normal Weight	*p*	Tukey’s Post Hoc Test
**Men**					
3-min step test	55.50 ± 10.16	57.65 ± 10.26	58.97 ± 10.70	<0.001 *	NW > OW > OB
1-min sit-up test	26.56 ± 10.00	28.45 ± 1.00	29.93 ± 10.29	<0.001 *	NW > OW > OB
Sit-and-reach test	20.43 ± 10.25	21.83 ± 10.52	22.05 ± 10.88	<0.001 *	NW, OW > OB
**Women**					
3-min step test	51.61 ± 13.06	54.86 ± 12.02	56.97 ± 11.00	<0.001 *	NW > OW > OB
1-min sit-up test	13.65 ± 10.06	15.54 ± 10.19	19.05 ± 10.36	<0.001 *	NW > OW > OB
Sit-and-reach test	26.29 ± 10.16	27.53 ± 10.69	27.91 ± 11.25	<0.001 *	NW > OW > OB

Abbreviations: NW, normal weight; OB, obesity; OW, overweight; SD, standard deviation; Values are expressed as means ± SD. * *p* < 0.05.

**Table 4:** Multivariate adjusted odds ratios (ORs) for overweight in relation to quartiles of health-related physical fitness measurements after adjustment for potential confounders.

Variables	Model 1 (Unadjusted)			Model 2 (Adjusted ^a^)		
	OR	95% CI	*p*	OR	95% CI	*p*
**Men**						
3-min step test						
< 51.14	1.367	1.267-1.474	<0.001 *	1.235	1.133-1.346	<0.001 *
51.14–56.59	1.302	1.204-1.408	<0.001 *	1.233	1.129-1.346	<0.001 *
56.60–64.29	1.183	1.098-1.274	<0.001 *	1.106	1.017-1.203	0.019 *
>64.29	Ref.	—	—	Ref.	—	—
Test for trend	*p* < 0.001 *			*p* <0.001 *		
1-min sit-up test						
<23.00	1.542	1.425-1.670	<0.001 *	0.896	0.806-0.996	0.041 *
23.00–29.99	1.430	1.324-1.545	<0.001 *	0.962	0.877-1.054	0.406
30.00–36.00	1.254	1.163-1.353	<0.001 *	1.004	0.922-1.093	0.926
>36.00	Ref.	—	—	Ref.	—	—
Test for trend	*p* < 0.001 *			*p* = 0.030 *		
Sit-and-reach test						
<15.00	0.914	0.843-0.990	0.027 *	0.748	0.684-0.819	< 0.001 *
15.00–21.99	1.014	0.936-1.097	0.740	0.887	0.811-0.970	0.008 *
22.00–30.00	1.032	0.957-0.990	0.410	0.931	0.856-1.013	0.096
>30.00	Ref.	—	—	Ref.	—	—
Test for trend	*p* = 0.017 *			*p* < 0.001 *		
**Women**						
3-min step test						
<49.73	1.605	1.482-1.738	<0.001 *	1.756	1.605–1.921	<0.001 *
49.73–55.20	1.185	1.090-1.288	<0.001 *	1.286	1.170–1.413	<0.001 *
55.21–62.50	1.145	1.056-1.241	0.001 *	1.174	1.072–1.413	0.001 *
>62.50	Ref.	—	—	Ref.	—	—
Test for trend	*p* < 0.001 *			*p* < 0.001 *		
1-min sit-up test						
<11.00	2.637	2.419-2.875	<0.001 *	1.361	1.217–1.521	<0.001 *
11.00–18.99	1.930	1.769-2.105	<0.001 *	1.322	1.194–1.464	<0.001 *
19.00–25.00	1.496	1.373-1.630	<0.001 *	1.207	1.097–1.328	<0.001 *
>25.00	Ref.	—	—	Ref.	—	—
Test for trend	*p* < 0.001 *			*p* < 0.001 *		
Sit-and-reach test						
<20.00	0.876	0.805-0.953	0.002 *	0.835	0.759–0.919	< 0.001 *
20.00–27.99	0.980	0.905-1.062	0.629	0.927	0.847–1.015	0.100
28.00–35.00	1.161	1.074-1.256	<0.001 *	1.118	1.024–1.221	0.013 *
>35.00	Ref.	—	—	Ref.	—	—
Test for trend	*p* < 0.001 *			*p* < 0.001 *		

Abbreviations: CI, confidence interval; OR, odds ratio; WHR, waist-to-hip ratio. * *p* < 0.05. ^a^ Adjusted for age, WHR, education, monthly income, marital status, self-reported health status, smoking status, and chewing betel nuts.

**Table 5:** Multivariate adjusted ORs for obesity in relation to quartiles of health-related physical fitness measurements after adjustment for potential confounders.

Variables	Model 1 (Unadjusted)			Model 2 (Adjusted ^a^)		
	OR	95% CI	*p*	OR	95% CI	*p*
**Men**						
3-min step test						
<50.56	2.340	2.147–2.551	<0.001 *	1.637	1.467–1.827	<0.001 *
50.56–56.24	1.696	1.556–1.850	<0.001 *	1.310	1.175–1.461	<0.001 *
56.25–63.38	1.267	1.159–1.385	<0.001 *	1.140	1.020–1.274	0.021 *
>63.38	Ref.	—	—	Ref.	—	—
Test for trend	*p* < 0.001 *			*p* < 0.001 *		
1-min sit-up test						
<22.00	2.482	2.264-2.722	<0.001 *	1.265	1.106-1.448	< 0.001 *
22.00–28.99	1.914	1.750-2.093	<0.001 *	1.174	1.044-1.321	0.007 *
29.00–35.00	1.606	1.470-1.754	<0.001 *	1.203	1.079-1.342	< 0.001 *
>35.00	Ref.	—	—	Ref.	—	—
Test for trend	*p* < 0.001 *			*p* = 0.003 *		
Sit-and-reach test						
<14.00	1.132	1.033–1.239	0.008 *	0.795	0.708–0.892	<0.001 *
14.00–20.99	1.273	1.164–1.392	<0.001 *	1.013	0.906–1.133	0.823
21.00–29.00	1.210	1.111–1.319	<0.001 *	0.999	0.898–1.112	0.992
>29.00	Ref.	—	—	Ref.	—	—
Test for trend	*p* = 0.007 *			*p* < 0.001 *		
**Women**						
3-min step test						
<49.45	2.896	2.621–3.200	<0.001 *	2.855	2.536–3.214	<0.001 *
49.45–55.20	1.450	1.302–1.615	<0.001 *	1.538	1.355–1.745	<0.001 *
55.21–62.07	1.105	0.988-1.236	0.079	1.101	0.967–1.255	0.147
>62.07	Ref.	—	—	Ref.	—	—
Test for trend	*p* < 0.001 *			*p* < 0.001 *		
1-min sit-up test						
<11.00	4.125	3.680–4.624	<0.001 *	2.387	2.051–2.778	<0.001 *
11.00–18.99	2.446	2.174–2.752	<0.001 *	1.910	1.658–2.200	<0.001 *
19.00–25.00	1.665	1.477–1.877	<0.001 *	1.459	1.272–1.673	<0.001 *
>25.00	Ref.	—	—	Ref.	—	—
Test for trend	*p* < 0.001 *			*p* <0.001 *		
Sit-and-reach test						
<20.00	1.067	0.955–1.192	0.249	0.843	0.739–0.960	0.010 *
20.00–27.99	1.343	1.210–1.492	< 0.001 *	1.102	0.975–1.246	0.120
28.00–35.00	1.405	1.265–1.560	< 0.001 *	1.198	1.060–1.355	0.004 *
>35.00	Ref.	—	—	Ref.	—	—
Test for trend	*p* = 0.659			*p* = 0.002 *		

Abbreviations: CI, confidence interval; OR, odds ratio; WHR, waist-to-hip ration. * *p* < 0.05. ^a^ Adjusted for age, WHR, education, monthly income, marital status, self-reported health status, smoking status, and chewing betel nuts.

## References

[1] Piernas C., Wang D., Du S., Zhang B., Wang Z., Su C., Popkin B.M. (2016). Obesity, non-communicable disease (NCD) risk factors and dietary factors among Chinese school-aged children. Asia Pac. J. Clin. Nutr..

[2] World Health Organization (2018). Obesity and Overweight. https://www.who.int/news-room/fact-sheets/detail/obesity-and-overweight.

[3] Wang Y.C., McPherson K., Marsh T., Gortmaker S.L., Brown M. (2011). Health and economic burden of the projected obesity trends in the USA and the UK. Lancet.

[4] Swinburn B.A., Sacks G., Hall K.D., McPherson K., Finegood D.T., Moodie M.L., Gortmaker S.L. (2011). The global obesity pandemic: Shaped by global drivers and local environments. Lancet.

[5] Stewart S.T., Cutler D.M., Rosen A. (2009). Forecasting the effects of obesity and smoking on US life expectancy. N. Engl. J. Med..

[6] Turer C.B., Brady T.M., de Ferranti S.D. (2018). Obesity, hypertension, and dyslipidemia in childhood are key modifiable antecedents of adult cardiovascular disease. Circulation.

[7] Westerterp K.R. (2017). Control of energy expenditure in humans. Eur. J. Clin. Nutr..

[8] Bryan A.D., Magnan R.E., Hooper A.E., Ciccolo J.T., Marcus B., Hutchison K.E. (2013). Colorado stride (COSTRIDE): Testing genetic and physiological moderators of response to an intervention to increase physical activity. Int. J. Behav. Nutr. Phys. Act..

[9] Herring M.P., Sailors M.H., Bray M.S. (2014). Genetic factors in exercise adoption, adherence and obesity. Obes. Rev..

[10] Bouchard C., Rankinen T., Timmons J.A. (2011). Genomics and genetics in the biology of adaption to exercise. Compr. Physiol..

[11] Ding D., Sallis J.F., Conway T.L., Saelens B.E., Frank L.D., Cain K.L., Slymen D.J. (2012). Interactive effects of built environment and psychosocial attributes on physical activity: A test of ecological models. Ann. Behav. Med..

[12] Foster S., Hooper P., Knuiman M., Christian H., Bull F., Giles-Corti B. (2016). Safe RESIDential environments? A longitudinal analysis of the influence of crime-related safety on walking. Int. J. Behav. Nutr. Phys. Act..

[13] Marcus B.H., Williams D.M., Dubbert P.M., Sallis J.F., King A.C., Yancey A.K., Franklin B.A., Büchner D., Daniels S.R., Claytor R.P. (2006). Physical activity intervention studies: What we know and what we need to know: A scientific statement from the American Heart Association Council on Nutrition, Physical Activity, and Metabolism Subcommittee on Physical Activity); Council on Cardiovascular Disease in the Young; and the Interdisciplinary Working Group on Quality of Care and Outcomes Research. Circulation.

[14] Sherwood N.E., Jeffery R.W. (2000). The behavioral determinants of exercise: Implications for physical activity interventions. Annu. Rev. Nutr..

[15] McGinn A.P., Evenson K.R., Herring A.H., Huston S.L. (2007). The relationship between leisure, walking, and transportation activity with the natural environment. Health Place.

[16] Marcus B.H., Dubbert P.M., Forsyth L.H., McKenzie T.L., Stone E.J., Dunn A.L., Blair S.N. (2000). Physical activity behavior change: Issues in adoption and maintenance. Health Psychol..

[17] Barlow C.E., Kohl H.W., Gibbons L.W., Blair S.N. (1995). Physical fitness, mortality and obesity. Int. J. Obes. Relat. Metab. Disord..

[18] Lee C.D., Blair S.N., Jackson A.S. (1998). US weight guidelines: Is it important to consider cardiorespiratory fitness?. Int. J. Obes. Relat. Metab. Disord..

[19] Wei M., Kampert J., Barlow C.E., Nichaman M.Z., Gibbons L.W., Paffenbarger R.S., Blair S.N. (1999). Relationship between low cardiorespiratory fitness and mortality in normal-weight, overweight, and obese men. JAMA.

[20] Gruberg L., Weissman N.J., Wakaman R., Fuchs S., Deible R., Pinnow E.E., Ahmed L.M., Pichard A.D., Suddath W.O., Satler L.F. (2002). The impact of obesity on the short-term and long-term outcomes after percutaneous coronary intervention: The obesity paradox?. J. Am. Coll. Cardiol..

[21] McAuley P.A., Blair S.N. (2011). Obesity paradoxes. J. Sports Sci..

[22] Uretsky S., Supariwala A., Singh P., Atluri P., Khokhar S.S., Koppuravuri H.K., Joshi R., Mandeva A., Rozanski A. (2010). Impact of weight on long-term survival among patients without known coronary artery disease and a normal stress SPECT MPI. J. Nuci. Cardiol..

[23] Romero-Corral A., Montori V.M., Somers V.K., Korined J., Thomas R.J., Allison T.G., Moodakam F., Lopez-Jimenez F. (2006). Association of bodyweight with total mortality and with cardiovascular events in coronary artery disease: A systematic review of cohort studies. Lancet.

[24] Lee C.D., Blair S.N., Jackson A.S. (1999). Cardiorespiratory fitness, body composition, and all-cause and cardiovascular disease mortality in men. Am. J. Clin. Nutr..

[25] Strasser B. (2013). Physical activity in obesity and metabolic syndrome. Ann. N. Y. Acad. Sci..

[26] Brook C.M., King D.S., Wofford M.R., Harrell T.K. (2005). Exercise, insulin resistance, and hypertension: A complex relationship. Metab. Syndr. Relat. Disord..

[27] Liao Y., Tsai H.H., Wang H.S., Ling C.P., Wu M.C., Chen J.F. (2016). Traveling by private motorized vehicle and physical fitness in Taiwanese adults. Int. J. Behav. Med..

[28] Liu C.M., Lin K.F. (2007). Estimation of VO2max: A comparative analysis of post-exercise heart rate and physical fitness index from 3-minute step test. JESF.

[29] Health Promotion Administration, Ministry of Health and Welfare Check Your Body Weight Every Day. https://www.hpa.gov.tw/Home/Index.aspx.

[30] American College of Sports Medicine—ACSM (2006). ACSM’s Guidelines for Exercise Testing and Prescription.

[31] Ayala F., Sainz de Baranda P., De Ste Croix M., Santonja F. (2012). Absolute reliability of five clinical tests for assessing hamstring flexibility in professional futsal players. J. Sci. Med. Sport..

[32] Ortega R.O., Grandes G., Sanchez A., Montoya I., Torcal J., PEPAF Group (2019). Cardiorespiratory fitness and development of abdominal obesity. Prev. Med..

[33] Brien S.E., Craig C.L., Katzmarzyk P.T., Gauvin L. (2007). Physical activity, cardiorespiratory fitness and body mass index as presictors of substantial weight gain and obesity. Can. J. Public Health.

[34] DiPietro L., Kohl H.W., Barlow C.E., Blair S.N. (1998). Improvements in cardiorespiratory fitness attenuate age-related weight gain in healthy men and women: The Aerobics Center Longitudinal Study. Int. J. Obes. Relat. Metab. Disord..

[35] Bailey M.W., Schubert C.M., Wentling V.J., Wurzbacher K.A., Czerwinski S.A., Demerath E.W., Siervogel R.M. (2003). Cardiorespiratory fitness during adulthood and CVD risks twenty years later: The FELS longitudinal Study. Med. Sci. Sports Exerc..

[36] Shook R.P., Hand G.A., Paluch A.E., Wang X., Moran R., Hebert J.R., Lavie C.J., Blair S.N. (2014). Moderate cardiorespiratory fitness is positively associated with resting metabolic rate in young adults. Mayo Clin. Proc..

[37] Motka P.K., Shah N.S. Abdominal muscle strength & its correlation with the BMI (Body Mass Index)—A survey in medical students. Proceedings of the 9th WCPT Africa Region Congress.

[38] Kamyabnia M., Jourkesh M., Keikha B.M. (2011). Comparison of physical fitness level among normal weight and obese female university students. Ann. Biol. Res..

[39] Kuk J.L., Saunders T.J., Davidson L.E., Ross R. (2009). Age-related changes in total and regional fat distribution. Ageing Res. Rev..

[40] Marcus R.L., Addison O., Dibble L.E., Foreman K.B., Morrell G., Lastayo P. (2012). Intramuscular adipose tissue, sarcopenia, and mobility function in older individuals. J. Aging Res..

[41] Roubenoff R. (2004). Sarcopenic obesity: The confluence of two epidemics. Obes. Res..

[42] Malina R.M., Beunen G.P., Classens A.L., Lefevre J., Vanden Eynde B.V., Renson R., Vanreusel B., Simons J. (1995). Fatness and physical fitness of girls 7 to 17 years. Obes. Res..

[43] Tokmakidis S.P., Kasambalis A., Christodoulos A.D. (2007). Fitness levels of Greek primary school children in relationship to overweight and obesity. Eur. J. Pediatr..

